# Rhinocerebral mucormycosis to the rise? The impact of the worldwide diabetes epidemic^☆^^☆☆^

**DOI:** 10.1016/j.abd.2020.06.008

**Published:** 2021-01-23

**Authors:** Erick Martínez-Herrera, Angélica Julián-Castrejón, María Guadalupe Frías-De-León, Gabriela Moreno-Coutiño

**Affiliations:** a Hospital Regional de Alta Especialidad de Ixtapaluca, Ixtapaluca, México; b General Hospital Dr. Manuel Gea González Calzada de Tlalpan, Tlalpan, Ciudad de México, México

**Keywords:** Diabetes mellitus, Epidemics, Mucormycosis, Opportunistic infections

## Abstract

The authors present seven cases of rhinocerebral mucormycosis associated to diabetes mellitus, which is a disease with epidemic proportions affecting individuals worldwide, particularly in developing countries, and which poses significant morbidity and mortality. Mucormycosis is an opportunistic fungal infection with high mortality and requires an invasive therapeutic approach to save the patient’s life with significant morbidity and sequelae, thus prevention is crucial.

## Introduction

Diabetes mellitus (DM) is one of the leading morbimortality diseases worldwide, with over 425 million affected individuals, particularly in low/medium income countries. Risk factors include an unhealthy sedentary lifestyle, diet high in refined sugar and fat, and genetic susceptibility^1^.

It is associated to numerous complications and comorbidities such as neutrophil dysfunction and impaired humoral immunity that predispose to fungal infections^2^.

Rhinocerebral mucormycosis is an opportunistic fungal infection of non-septate hyaline hyphae which are ubiquitous saprophytes that are easily isolated from decomposing vegetation and high fructose fruits, soil, manure, and human orifices^3^.

**Figure 1:**
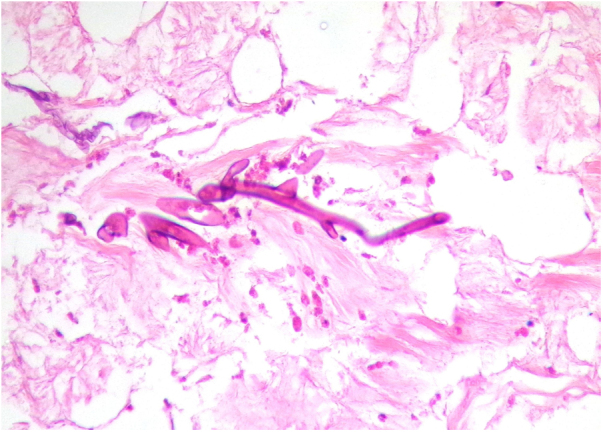
Fungal structures. Thick hyphae are highlighted with PAS stain, next to a small vessel (PAS stain, ×40).

**Figure 2:**
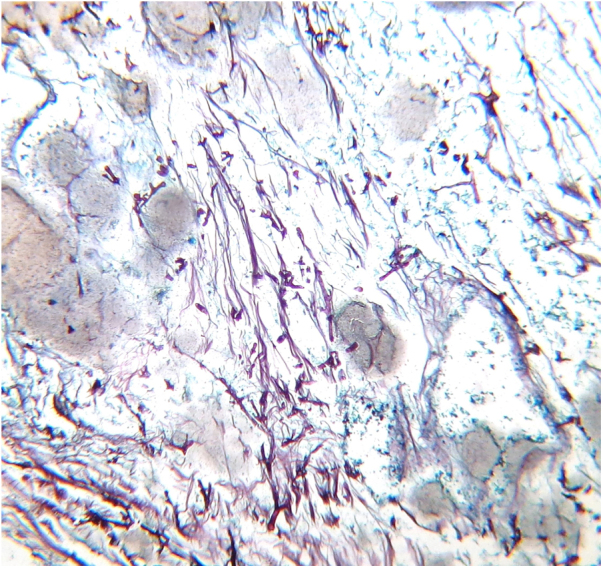
Numerous thick hyphae are observed (Grocott-Gomori stain, ×10).

Despite adequate treatment, this infection has high mortality (40%–63%), and its prevalence has increased, probably due to the growing number and higher survival rates of immunosuppressed patients^4^, ^5^. Most infections are life threatening, as the fungus has particular affinity for vascular walls that cause irreversible damage through thrombosis and ischemic necrosis. Figure 1, Figure 2 present biopsy specimens taken from patients for diagnosis, and the fungus can be seen invading the normal tissue.

The well identified predisposing factors are ketoacidosis by decompensated diabetes mellitus, advanced kidney failure, drug abuse, immunosuppressive treatment, or any other disease that causes neutropenia or polynuclear neutrophil deficit^4^, ^5^, ^6^, ^7^.

Initially, most patients complain of symptoms similar to acute bacterial sinusitis, but rapid deterioration occurs with alterations in the visual acuity, facial edema, and necrotic areas, which are the hallmarks of the disease, either on the face or intraorally. Orbit involvement occurs in 66%–100% of the cases and any contiguous region may also be involved^5^.

Timely diagnosis is fundamental for a positive outcome, so treatment with amphotericin B should be initiated on clinical presumption. Also, aggressive surgical debridement is fundamental, as removing the necrotic areas will reduce the fungal burden and will also facilitate the antifungal’s action^3^, ^4^. Amphotericin B improves survival rate up to 55%, adding to the results of surgical debridement. Another option is posaconazole and hyperbaric oxygen^3^, ^5^, ^7^.

## Cases report

**Figure 3:**
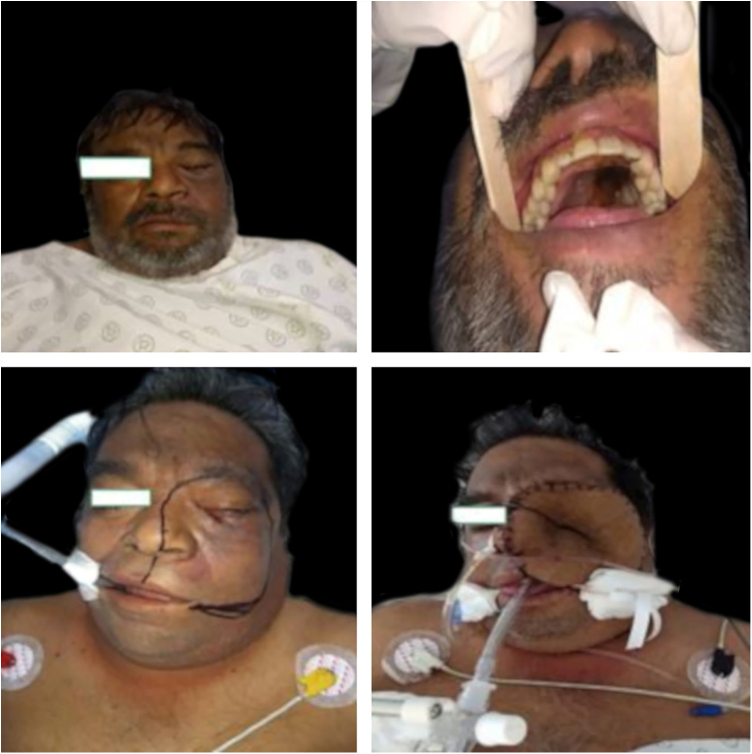
Clinical picture of a patient before and after extensive surgery.

**Figure 4:**
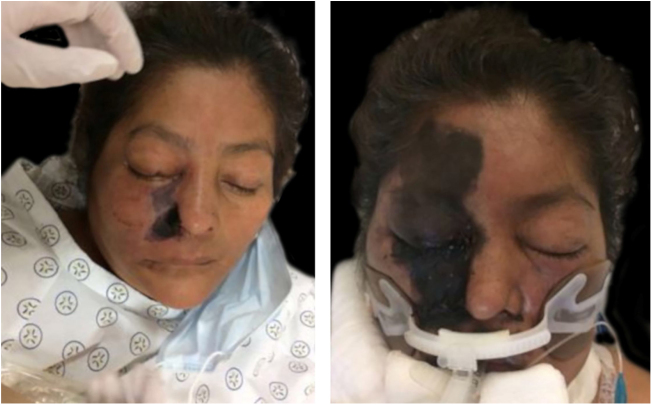
Necrotic area and its rapid progress.

The authors present seven cases of diabetic patients that attended this tertiary care hospital with rhinocerebral mucormycosis (Table 1). They were three males and four females, with ages ranging from 18–64 years (Figure 3, Figure 4). One of them was referred to another hospital because of lack of space, another did not accept surgery, and another died before the surgery was performed. Of the four patients who underwent surgery, two died in the postsurgical period and the other two were discharged for improvement after several weeks. All of them received amphotericin B as part of the treatment, including those who did not undergo surgery.

**Table 1:** Patient data and evolution.

Patient	Sex/age	Initial involved area	Surgery (qx)	Evolution	Result	Other diseases
1	M/46	Eye, palate	qx	Surgical complications	Voluntary discharge	
2	F/18	Maxillary area	qx		Released for improvement	Obesity
3	F/50	Right face edema	No qx	Died before surgery		HAS
4	M/45	Right eye, hard palate	No qx	Did not accept treatment option	Died one monthlater	
5	F/64	Hard palate, Left eye		Referred, lost to follow-up		
6	F/41	Left side of the face	qx	Complications and died		Nephrotic infection
7	M/51	Left eye, cervical pain	qx		Discharged for improvement

All of the patients acquired the fungal infection prior to the hospitalization, except for one patient who was admitted to the hospital for a nephrotic infection that resulted in nephrectomy; after several days she developed mucormycosis symptoms.

Of the three patients who died, two of them had metabolic dyscontrol and all presented ketoacidosis at some point during the disease.

## Discussion

The diagnosis of mucormycosis requires clinical suspicion and quick action, as the fungal invasion of the vessels advances rapidly. Its diagnosis is considered an emergency. Ideally, it must include mycological diagnosis with a direct exam and a culture that grows in 24 hours. Also, to determine the extent of the damage, imaging tests are of great help, such as tomography. Surgical debridement provides a chance to cure, and material for histopathological confirmation. The main differential diagnosis is invasive aspergillosis.

DM is a global burden with a rise in prevalence and incidence during the last decade, and besides being directly related to death, it is also very associated to serious morbidity and disability.

For example, in the Mexican population, since the year 2000, DM is the leading cause of death among women and the second in men, representing 15% of deaths. Up to 90% of the cases are related to obesity, and 72% of Mexican adults are obese^8^. Thus, the public health system must simultaneously address these two diseases as an epidemiological emergency. This scenario may also be observed in other countries, particularly those of Latin America that have similarities to Mexico, which must also be aware of this risk.

These seven cases were related to this metabolic disease, supporting that mucormycosis is an opportunistic fungal infection that affects critically ill patients of any age and with any kind of immunosuppression, particularly those living with diabetes.

These cases are an example of what may become a more and more frequent diagnosis in the near future if this obesity epidemic is not reverted or at least slowed down.

## Financial support

None declared.

## Authors’ contributions

Erick Martínez-Herrera: Data collection, analysis, and interpretation; effective participation in research orientation.

Angélica Julián-Castrejón: Effective participation in research orientation; intellectual participation in propaedeutic and/or therapeutic conduct of studied cases.

María Guadalupe Frías-De-León: Data collection, analysis, and interpretation; intellectual participation in propaedeutic and/or therapeutic conduct of studied cases.

Gabriela Moreno Coutiño: Approval of the final version of the manuscript; study conception and planning; critical review of the literature; critical review of the manuscript.

## Conflicts of interest

None declared.
